# Disproportional Representation of Primates in the Ecological Literature

**DOI:** 10.1371/journal.pone.0080763

**Published:** 2013-12-10

**Authors:** Eckhard W. Heymann, Dietmar Zinner, Jörg U. Ganzhorn

**Affiliations:** 1 Abt. Verhaltensökologie & Soziobiologie, Deutsches Primatenzentrum, Göttingen, Germany; 2 Abt. Kognitive Ethologie, Deutsches Primatenzentrum, Göttingen, Germany; 3 Abt. Tierökologie, Zoologisches Institut, Universität Hamburg, Hamburg, Germany; The University of Texas at San Antonio, United States of America

## Abstract

We address the question why papers dealing with the ecology of primates are so sparsely represented in the general ecological literature. A literature analyses based on entries in Web of Science and PrimateLit reveals that despite a large number of papers published on primates in general and on the ecology of primates, only a very small fraction of these papers is published in high-ranking international ecological journals. We discuss a number of potential reasons for the disproportion and highlight the problems associated with experimental research on wild primates and constraints on sample size as major issues.

## Introduction

Primatological studies have played a major role in the development of conceptual frameworks in evolutionary ecology. The comparative approach of J. H. Crook on the social organization of birds and primates was instrumental to the development of the fields of “socio-biology” and “behavioral ecology” in general [1], [2]. His work stimulated a number of primatological papers that contributed to the development of general ecological questions (e.g., [3], [4]). Since then, the comparative approach seems to have shifted away from primate ecology, and primate ecology seems to have lost importance for the academic community at large, while analyses of social systems still remain prominent (e.g., [5]). Many ecological studies in primatology are designed or considered “case studies” rather than studies of general interest that contribute to the understanding of ecological and evolutionary questions in general. This perception or reality has important consequences as, first, academic funding agencies are reluctant to fund case studies, and second, the scientific community and primatologists themselves are unaware of the wealth of information for many distinct species (probably unmatched by any other taxon) and the potential to contribute to the understanding of general evolutionary questions in ecology. With the present analysis we wanted to test whether this perception on the lack of importance of primatological studies in the ecological literature is justified.

This perceived lack of importance is even more intriguing when considering that the number of publications on primate ecology is very high when compared to other taxa with a much larger number of species. A search in Web of Knowledge^SM^ v.5.9 (search date: 18 June 2013) with terms “primate* and ecology”, “mammal* and ecology”, “bird* and ecology” and “insect* and ecology” yielded 1646, 5947, 8979 and 9749 hits, respectively. Comparing these figures with the number of species (primates: 479, mammals: ∼6000, birds: ∼10.000, insects: >1.000.00) reveals an obvious disproportion: in relation to the number of species, there are many more papers on primates than on the other taxa.

Therefore, in this paper we examine the following questions:

1. Which fraction of papers published on primates (independent of the type of publication, i.e. journal articles, book chapters etc.) deals with aspects of ecology?
2. Which fraction of papers on primate ecology is published in high-ranking ecological journals?
3. Which fraction of papers published in high-ranking ecological journals deals with primates and how does this compare with other taxa?

We then discuss potential reasons for the underrepresentation of primate papers in ecological journals.

## Methods

To examine the significance of ecological research questions in primatology, we searched in PrimateLit (http://primatelit.library.wisc.edu) with “keyword  =  ecology and feral”. The term “feral” was used to exclude papers dealing with “lab ecology”, a keyword used in PrimateLit for papers dealing with housing of and environmental enrichment for captive primates. We restricted this search to the period 1980–2009 for two reasons: (a) some of the currently high-ranking ecological journals only started in the 80 s or 90 s; (b) PrimateLit ceased to be updated after November 2010. We plotted 5-year running means of the number of hits per year against the time in years.

To examine the representation of papers dealing with primate ecology in ecological journals, we performed two different analyses. First, we searched in PrimateLit, with “keyword  =  ecology and feral” and “publication  =  <journal name>” and restricted this search to the period 1980–2009. We used the following journals on the basis of their scope (which should make a journal potentially feasible for submitting primate ecology papers) and their impact factors: Ecology, Ecology Letters, Functional Ecology, Journal of Animal Ecology, Journal of Ecology, Oecologia, Oikos. We then determined the total number of articles dealing with any taxon or topic in the journals mentioned above by searching the Web of Knowledge^SM^ [v.5.9] with the journal name in the field “publication name” (search date: 15 April 2013). On the basis of these search results, we calculated the percentage of papers from the search in PrimateLit that were published in the above mentioned journals. We also examined, on the basis of values summed up over 5-year periods, whether there is a temporal trend in the representation of primate ecology papers in ecological journals.

Second, we compared the primate data with other taxa, namely insects, all classes of vertebrates, and major orders of mammals. We searched the Web of Knowledge Knowledge^SM^ v.5.9 (search date: 18 June 2013) first with the search term “<taxon> and ecology” (e.g. “bird* and ecology”) in the field “topic” and then with this term plus the journal name in the field “publication name”. The search period comprised 1945–2013. We then calculated, for each taxon and over the entire search period, the percentage of papers in the above mentioned ecological journals. To estimate how primate papers perform in comparison to other taxa, we calculated the mean percentage and SD over all taxa except primates and compared this mean to the value for primates with a single sample t-test in Statistica 10.0 [6].

## Results

### Significance of ecological research questions in primatology

**Figure 1:**
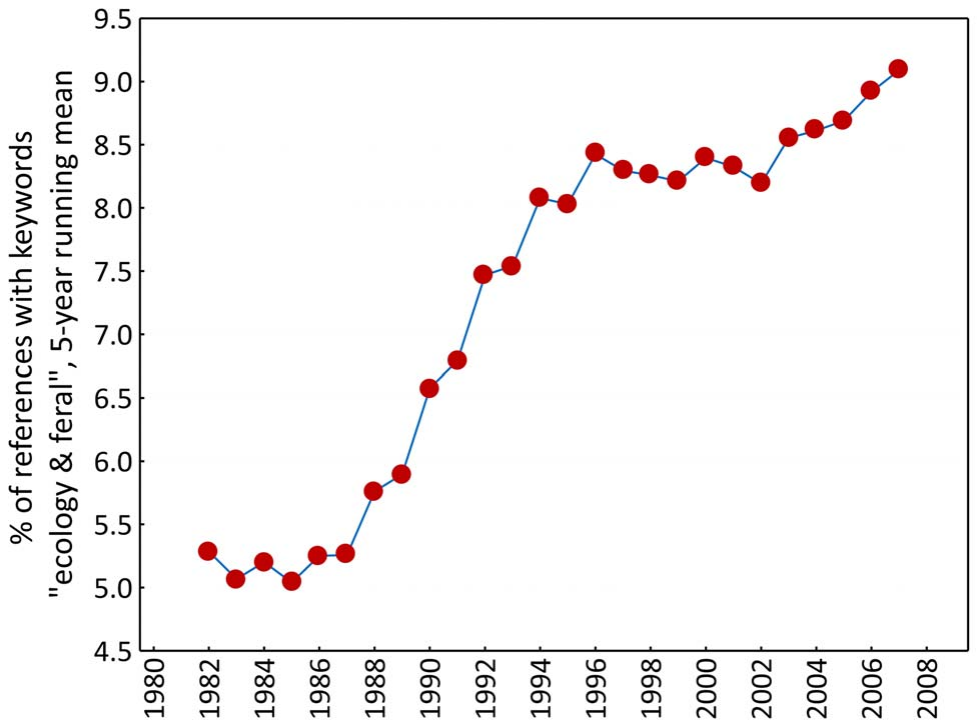
Temporal trend in the percentage of primatological publications dealing with aspects of primate ecology over the period 1980–2009. Dots represent 5-year running means; therefore, 1980, 1981, 2008 and 2009 are not represented by a dot.

Over the period 1980–2009, 7.6% of papers listed in PrimateLit dealt with aspects of primate ecology. The percentage almost doubled between 1980 and 2009, with a steep increase starting around 1990 (Fig. 1).

### Representation of primate papers in ecological journals

**Figure 2:**
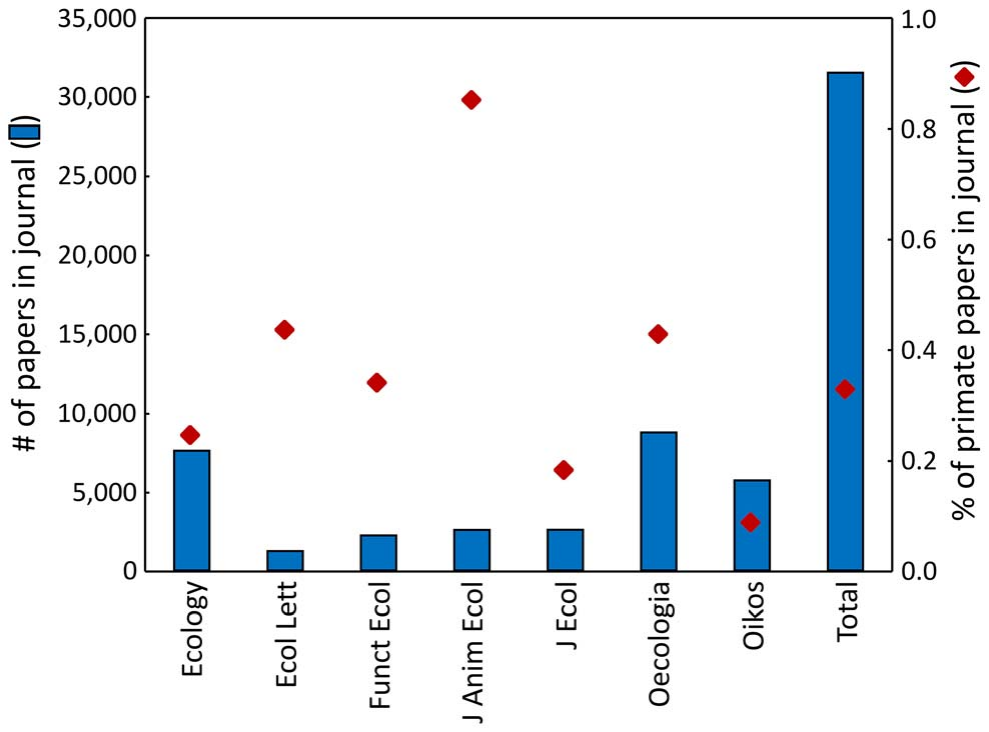
Total number of papers published in different ecological journals and percentage of these papers that relate to primates. Ecol Lett  =  Ecology Letters, Funct Ecol  =  Functional Ecology, J Anim Ecol  =  Journal of Animal Ecology, J Ecol  =  Journal of Ecology.

Papers dealing with aspects of primate ecology are only marginally represented in high-ranking ecological journals, accounting on average for only 0.3% of all papers in these journals (Fig. 2). For the period 2010–2013, this remains unchanged (0.3% of a total of 4980 papers).

**Figure 3:**
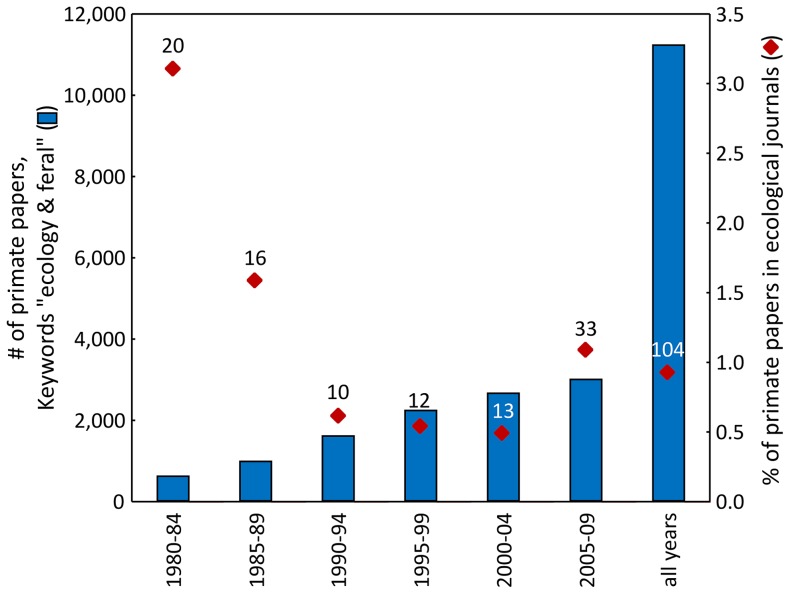
Number of primate papers over 5-year periods and percentage of primate papers in ecological journals. Numbers above diamonds indicate the total number of primate papers in ecological journals in the respective period.

The representation of papers dealing with primate ecology in ecological journals dropped drastically from >3% in 1980–84 to the currently low value with some fluctuation over time, despite the strong increase in the total number of papers dealing with primate ecology (Fig. 3). The absolute number of primate papers in ecological journals varied relatively little between 1990 and 2004 (10–20 papers/5 year period), and increased to 33 in the period 2005–2009. The total number of primate papers with “keyword  =  ecology and feral” increased from 644 (1990–94) to 3027 (2005–09). Over the entire 30-year period, the percentage of primate ecology papers published in ecological journals is below 1%.

### Comparison with other taxa

**Figure 4:**
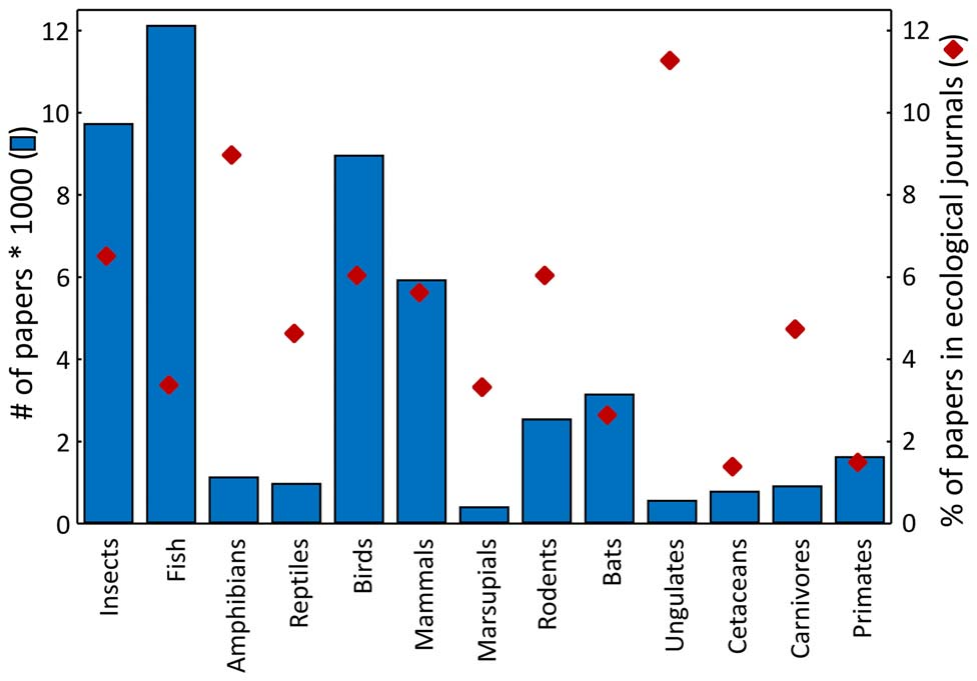
Number of papers on the ecology of insects, different classes of vertebrates and major orders of mammals, and the percentage of these papers that are published in high-ranking ecological journals.

Compared to other taxa, primates are poorly represented in ecological journals. The proportion of papers that matched the search term “primate* and ecology” (1.5%) was lower than for most other taxa except for cetaceans that had an even lower value (1.4; Fig. 4). The mean percentage ± SD over all taxa is 5.1±2.8, and only primates and cetaceans lie outside one SD from the mean. The percentage for primates (1.5) is significantly smaller than the mean percentage over all other taxa (5.4±2.7; t = 4.878, d.f.  = 11, p = 0.000487).

## Discussion

Our analyses revealed that despite a strong increase in the number of papers dealing with primate ecology over the last decades, there is a disproportionately low representation of such papers in general ecological journals. The general increase in primatological papers was not matched by a similar increase in the number of primate papers published by ecological journals. Their percentage actually dropped (Fig. 3). Furthermore, the representation is similar to the representation of cetaceans and considerably lower than for most other taxa. There are several potential explanations for this which we discuss below:

- “Bad ecologist” hypothesis

Many primatologists may be interested in primates per se rather than in ecological problems and lack a sound theoretical background in general ecology. Thus, they may either not attempt publication outside the primatological circle, or they may not ask the “right” questions, or they cannot put their findings into a perspective that makes the results of their research interesting for a broader ecological readership. This is perhaps related to the history of primate ecological research. As Clutton-Brock [7, p. vii] stated in the preface to one of the first books dedicated to primate ecology, “interest in primate ecology has been stimulated by investigation of the adaptive significance of social systems”. Furthermore, as Janson [8, p. 521] pointed out, “field primatology emerged largely from a descriptive anthropological tradition” which led to primate field studies focusing on a more descriptive primate ecology (“The diet of the …”, “Habitat use in a population of …” etc.). This is not necessarily “bad ecology”, but it clearly makes findings less appealing to a wider, ecologically interested audience. The relatively minor fluctuation in the total number of primate papers in ecological journals over the years, despite an almost 5-fold increase in the number of primate papers with keyword “ecology” and “feral” from 1990 to 2009, probably reflects this rather descriptive approach in primatology.

Carmel et al. [9] recently analysed trends in ecological research by examining the topics and approaches of papers published in all ecological journals and in “core ecological journals” (partially overlapping with our journal list) between 1980 and 2010. They found that a majority of studies deals with single species (71% and 66% of papers in all journals and core journals, respectively) and use observational approaches (59% and 45%). Therefore, the principal setup of most primate studies – observational on single species – should not preclude publication in ecological journals (although the percentage of observational studies in core ecological journals is considerably lower than in all ecological journals).

- Lack of experimental confirmation

Many research questions in ecology require experimental work to confirm observational findings and to test hypotheses. Lack of experimental confirmation of findings may result in lower acceptance of primate ecological research to ecologists who advocate more rigid experimental approaches. However, many experiments are impossible with primates on ethical and/or on practical grounds.

### Ethical limitations

Primatologists have used playbacks of conspecific or allospecific vocalizations [10]–[12], platforms with food and/or sensory stimuli [13], [14], cognitive test apparatuses [15], [16], and provisioning with tools [17], [18] to manipulate the behaviour and study the cognitive and sensory capabilities of primates. These kinds of experiments raise few ethical concerns, if any. However, many ecological experiments may require much stronger manipulation. E.g., to test whether a species exerts a competitive effect on another species or whether the lack of pollinators or seed dispersers affects plant populations requires the removal of complete groups or populations. For primate ecology studies, this would be absolutely unacceptable for ethical reasons.

As a proxy, pseudo-experimental situations where primate species have been extinguished, occur in reduced population densities due to hunting, or are absent for other reasons can provide relevant information. A comparison of hunted and non-hunted sites in Amazonia revealed competitive effects of large-sized on medium-sized primate species [19]. Similarly, a comparison of sites with low and high hunting pressure demonstrated that the lack of primate seed dispersal affects patterns of plant recruitment [20]. The absence of spider monkeys resulted in higher fruit set in a population of *Symphonia globulifera* compared to a population where these primates heavily preyed upon flowers of this plant [21].

Given that the majority of primates are group-living [5] experiments that require the temporal removal or isolation of individuals from a group are also ethically problematic (apart from practical problems). Experiments like those of Kawai [22] where entire groups were captured and released at a different place would no longer receive approval. Consequently, ecological experiments that require temporal removal of animals from their natural setting have been performed with solitary foragers like mouse lemurs, *Microcebus* [23], [24], but not with group-living primates.

### Practical limitations

- Sample size limitations

The majority of primates live in groups that range together and show a high degree of co-ordination and synchronization of activities [25], [26]. Individuals within such groups thus cannot be considered as statistically independent samples. Observing a sufficiently large number of different groups to obtain high statistical power is in most cases practically impossible (due to limitations of funding, time constraints, limited size of study areas etc.).

Sample size can also be limited due to the high behavioural and ecological flexibility and high intra-specific and intra-population variability of most primates (see (ii)).

- Flexibility and variability

Most primates are behaviourally and ecologically highly flexible [27]–[29]. This requires either many replica or the control of a large number of potentially or actually confounding variables; something that is rarely done in primate ecology [8]. Most primates also have slow life histories and long generation times [30] which can introduce an additional major source of variation [31]. Together with the issue of sample size limitations discussed above, this results in low statistical power.

- Primate intelligence and cognition

Primates – by virtue of their higher cognitive capabilities compared to most other animals – can rapidly habituate to experimental stimuli and settings. They may even manipulate or destroy the experimental setup (see [32] for an example from captive primates). Even if planned very well and based on previous knowledge and experience, experiments may fail, simply because primates respond in an unanticipated manner. Janson [8] provides first-hand examples on failures of ecological and behavioural field experiments with primates. E.g., a setup that had worked very well with some groups, did not work with another group, or experiments turned out not “to fit within the lifestyle” [8, p. 531] of the animals. Experimental feeding platforms may not be attractive enough if alternative food resources become available, a factor that may be uncontrollable in the often large home ranges and complex tropical habitats of primates.

From all other taxa, practical problems, particularly for experimentation, and ethical concerns are probably most similar in cetaceans. The low representation of this taxon in high-ranking ecological journals supports our hypotheses outlined above.

There are a few other conceivable hypotheses that may apply.

- “Boring primates” hypothesis: Most primates live in tropical forest and occupy only a few, relatively similar niches. Therefore, ecological questions are similar for different primate species.
- “Geographic origin of researcher” hypothesis: Many researcher study ecology of species living in their home countries. Since still most researchers come from northern industrialized countries, species living in these regions could be overrepresented in the ecological literature.
- “Inbreeding” hypothesis: Primatologists may tend to publish their results – even if they could be of more general interest – in taxon-specific journals. Conversely, editors of general-interest ecology journals may be more willing to decide that ‘there are other more appropriate journals for this manuscript’ simply because in fact there are many primate journals relative to any other comparable order of animals (e.g. there is no ‘Journal of Woodpeckerology’). However, the latter seems to be unlikely. This can be illustrated by the case of the representation of papers on birds and rodents. Both taxa have a similar representation in ecological journals (Fig. 4), but while there are 130 or more ornithological journals [33], we are aware of only one journal dedicated to rodents.

### Conclusions

We have identified some potential causes for the low representation of primate papers in the general ecological literature, but do not pretend that this is an exhaustive listing. Conceptual problems are probably the easiest to overcome by modifying the curricula in primatology classes. Most practical problems could be overcome in an ideal world with less restricted funding and less time pressure for publications. However, in the real world, these problems constrain ecological research on primates and consequently may lead to results of primate ecological research being less attractive for a general ecological audience. Nevertheless, we hope that this paper stimulates discussions, both within the primatological community and between primate ecologists and general ecologists. Primate ecology has the potential to make important contributions to general ecology, particularly with regard the long-term ecological variation [34] and consequences of personality for ecological interactions and processes [35].
